# Pre-clinical medical student experience in a pediatric pulmonary clinic

**DOI:** 10.3402/meo.v20.28654

**Published:** 2015-11-04

**Authors:** Thomas G. Saba, Marc B. Hershenson, Manuel Arteta, Ixsy A. Ramirez, Patricia B. Mullan, Sonal T. Owens

**Affiliations:** 1 Department of Pediatrics and Communicable Diseases, University of Michigan Medical School, Ann Arbor, MI, USA; 2 Department of Learning Health Sciences, University of Michigan Medical School, Ann Arbor, MI, USA

**Keywords:** early clinical experience, pediatrics, undergraduate medical education, pre-clinical education

## Abstract

**Objective:**

Our objective was to evaluate the educational value of introducing pre-clinical medical students to pediatric patients and their families in a subspecialty clinic setting.

**Methods:**

First- and second-year medical students at the University of Michigan seeking clinical experience outside of the classroom attended an outpatient pediatric pulmonary clinic. Evaluation of the experience consisted of pre- and post-clinic student surveys and post-clinic parent surveys with statements employing a four-point Likert scale as well as open-ended questions.

**Results:**

Twenty-eight first-year students, 6 second-year students, and 33 parents participated in the study. Post-clinic statement scores significantly increased for statements addressing empathic attitudes, confidence communicating with children and families, comfort in the clinical environment, and social awareness. Scores did not change for statements addressing motivation, a sense of team membership, or confidence with career goals. Students achieved their goals of gaining experience interacting with patients, learning about pulmonary diseases, and observing clinic workflow. Parents felt that they contributed to student education and were not inconvenienced.

**Conclusions:**

Students identified several educational benefits of exposure to a single pediatric pulmonary clinic. Patients and families were not inconvenienced by the participation of a student. Additional studies are warranted to further investigate the value of this model of pre-clinical medical student exposure to subspecialty pediatrics.

Since William Osler brought students to the bedside, direct patient contact has played an essential role in medical education. Medical school curricula have traditionally been designed to bring students to the bedside only after they have learned the basics of pathophysiology in the classroom. Over the last 30 years, however, there has been a trend to integrate clinical experiences into pre-clinical education. The Carnegie Foundation for the Advancement of Teaching report, published in 2010 (1), emphasized the need to integrate formal knowledge with clinical experience.

Numerous studies have investigated the educational outcomes of exposing students to patients prior to traditional clerkship rotations. Best Evidence Medical Education (BEME) Collaboration systematic reviews by Dornan et al. (2) and Yardley et al. (3) identified several important educational outcomes for students exposed to patients early and paved the way for curricular modifications promoting early clinical experience (4, 5) and a vigorous re-evaluation of pre-clinical medical education.

The majority of studies performed to date were longitudinal programs for first- and second-year students in primary care/community settings where students interacted with adult patients. Interactions with children with chronic diseases and their families, however, can present a unique clinical learning environment. For medical schools to justify pre-clinical pediatric patient contact, empirical research on the impact on students, patients, and their families is needed.

For several years, the Pediatric Pulmonologists at the University of Michigan have taken on the practice of inviting pre-clinical students to observe clinical care in pediatric pulmonary outpatient clinics. Although anecdotally, we have received positive feedback from students, we sought to more systematically evaluate this practice. The objective of this study was to evaluate pre-clinical medical student exposure to children with chronic medical conditions from the perspectives of both the students and the patients’ families.

## Methods

Subjects included first- and second-year medical students at the University of Michigan Medical School who volunteered to shadow a pediatric pulmonologist for a half day in his or her outpatient pediatric pulmonary clinic. Each medical class is composed of 170 students. All students had received didactic anatomy and physiology teaching but nearly none had any experience in interacting with actual patients. Students observed the doctor–patient encounter and performed a very basic pulmonary exam directed by the physician. Physical contact was considered to be an important part of this experience. Some students spent a few minutes alone with the patient and family when the physician was occupied with another patient. The student interaction with the family, therefore, did not extend the length of the clinic visit. Families were generally accustomed to the practices of a teaching hospital since they often used other hospital services in which learners participated. Families were asked to provide feedback. Internal Review Board exemption was obtained prior to subject recruitment.

**Fig. 1:**
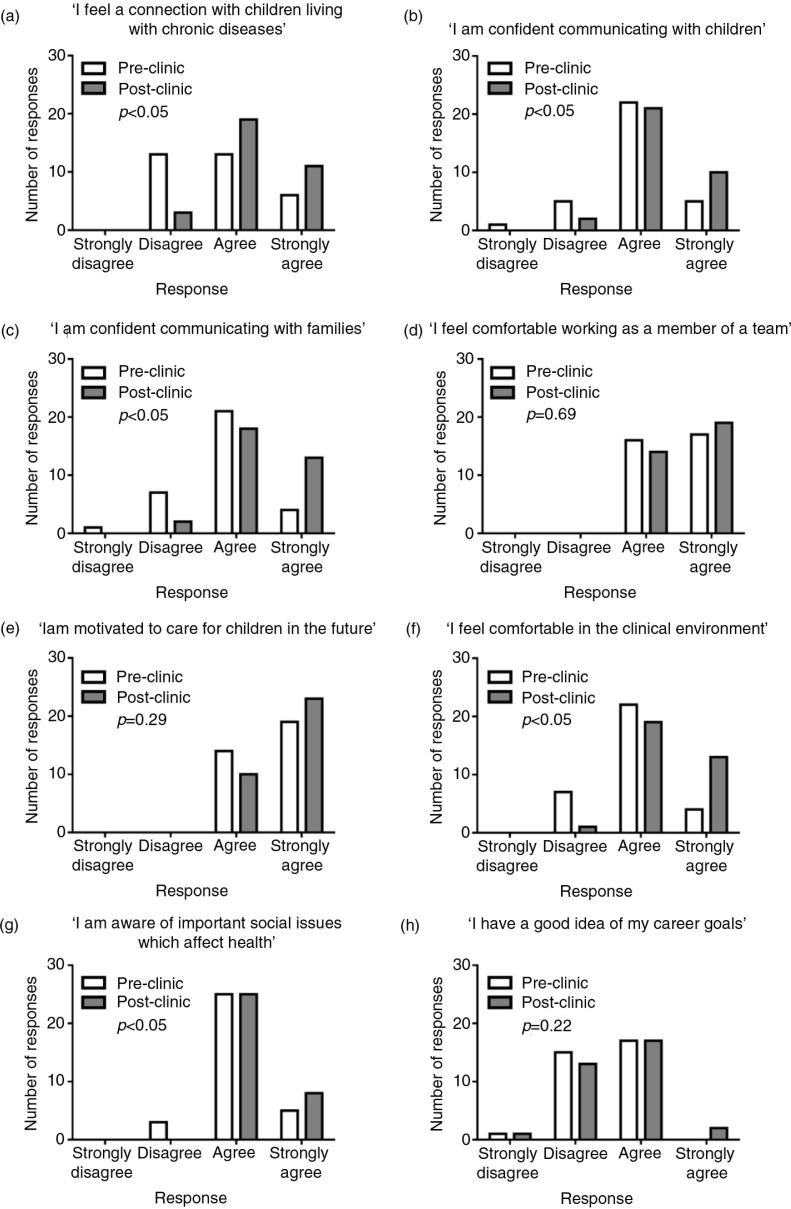
Pre- and post-clinic student responses to eight statements. Differences between pre- and post-clinic responses were statistically significant for statements a, b, c, f, and g. There was no difference in pre- and post-clinic scores for statements d, e, and h. *p*<0.05 denotes statistical significance.

Students were asked to complete a pre-clinic survey consisting of eight statements addressing their attitudes towards working with real pediatric patients (Fig. 1), as well as open-ended questions. These statements were derived from recent BEME reviews on early clinical experience (2, 3) and vetted independently by a group of Pediatric Pulmonary physicians and physicians enrolled in a Medical Education training course at the University of Michigan. Statement responses employed a four-point Likert scale from 1 to 4 corresponding to ‘Strongly disagree’, ‘Disagree’, ‘Agree’ and ‘Strongly agree’, respectively. At the end of the clinic, students completed a post-clinic survey consisting of the same eight statements and open-ended questions. Families that met with the student were asked to respond to three statements: 1) interacting with the student allowed me to contribute to this student's education, 2) interacting with the student was a pleasurable experience and 3) interacting with the student was inconvenient. Responses to these statements employed the same four-point Likert scale. Parents were also asked open-ended questions.

For each student, we calculated the difference between the pre- and post-clinic survey responses for each statement on the survey. The sign test on the difference compares the median difference between pre- and post-clinic responses of every statement to 0 (no difference). We did not control for multiple comparisons due to the small sample size and scope of the study. A two-tailed significance level of 0.05 was employed. Two coders independently reviewed students’ and parents’ responses to open-ended questions and identified common themes.

## Results

Thirty-four students (28 first-year and 6 second-year) participated in the program between February 2013 and March 2014. Forty parents offered feedback. We had a 100% participation rate for all surveys administered. Analyses of parent responses included all (40) parents’ written comments to the open-ended questions and the statement responses of 33 parent surveys.

Overall, students demonstrated more agreement with statements in the post-clinic surveys (Fig. 1). The difference between pre- and post-clinic scores met the pre-determined level of significance for five out of the eight statements, which reflected students’ sense of connection with children living with chronic diseases, confidence communicating with patients and families, comfort in the clinical environment and awareness of social determinants of health (Fig. 1a–c,f,g). The difference between pre- and post-clinic scores was not statistically significant for three out of the eight statements, which elicited comfort working as a member of a team, motivation to care for children in the future, and confidence with career goals (Fig. 1d,e,h). However, all students either agreed or strongly agreed that they were motivated to care for children and felt a sense of team membership on the pre-clinic surveys.

Prior to participating in the clinical experience, students were asked, ‘What are your learning objectives?’ from which five themes emerged: students wanted to 1) gain a better understanding of pediatric pulmonary diseases, 2) observe first-hand the clinic flow and doctor–patient interactions, 3) gain experience talking to families, 4) learn about differences between pediatric and adult care, and 5) discover if they had an interest in pursuing a career in pediatrics.

At the end of the clinic, students were asked ‘What specifically have you gained from this educational experience’, from which three themes emerged: students 1) gained greater comfort and confidence interacting with children and their families, 2) learned about disease pathophysiology, testing, and treatment, and 3) gained an appreciation for multi-disciplinary care and the typical workday of a pediatric pulmonologist. Students were also asked, ‘What were some of the challenges you faced today and how might you modify the program?’ to which they admitted to feeling nervous and unprepared.

Additional comments from students were universally positive. Comments included: ‘The chance to talk to patients and ask questions about their health was a unique and pleasant surprise’, ‘I learn best through experience, so that is the main reason I want as much clinical exposure as possible at this stage in my career’, and ‘I leave here today having learned more than many of my lectures have taught me’.

Ninety-seven percent of parents agreed or strongly agreed that they contributed to the student's education and found it to be a pleasurable experience, and 97% disagreed or strongly disagreed that it inconvenienced them. There was only 1 questionnaire out of 33 which offered consistently negative feedback. Unfortunately, no comments were provided by this family.

Parents were asked ‘How did the medical student affect your family's visit to the pulmonary clinic?’ Parents generally perceived it to be a positive experience for their families and did not think it affected the clinic visit. They recognized their value in training future doctors.

## Discussion

In this study, both quantitative and qualitative data were used to evaluate a practice of inviting pre-clinical students to a pediatric subspecialty clinic. Students identified some positive educational outcomes even with the brief clinical encounter and it did not cause significant inconvenience to families.

Very few studies in the early clinical experience literature have looked specifically at exposure to pediatrics. Three studies have identified positive outcomes when students interacted with healthy children (6–8) and only one study by Satran et al. (9) exposed students to pediatric patients in hospital and ambulatory care settings. This study provided insight into the educational benefits of exposing pre-clinical students to children with chronic diseases and their families. The students in this study gained skills and confidence, which would potentially help ease the transition from didactic to clinical learning.

Students’ motivation to care for children, sense of team membership, or insight into their career goals did not change as a result of this experience. It is important to note, however, that all students either agreed or strongly agreed that they were motivated to care for children and felt a sense of team membership even before their clinical experience. Prior studies of longitudinal early clinical experiences have shown a positive impact on students’ career choices particularly in family medicine and rural/community health (10). In this study, a single exposure did not influence students’ career choices, as would be expected.

Feedback from parents provided valuable insight into the feasibility of this model. Prior studies have identified that patients are generally satisfied with interactions with first- and second-year medical students and recognize the importance of teaching (11, 12). In a study looking at families’ feedback of student involvement in pediatric care, Passaperuma et al. (13) identified that families reported greater comfort with students in third- and fourth-year and were neutral to first-year students. In our study, however, the vast majority of families thought that interacting with first- and second-year students was a pleasurable experience and appreciated the opportunity to contribute to their education. A more thorough investigation of families’ perceptions of undergraduate medical education was not sought. Parent satisfaction in this clinical scenario cannot necessarily be extrapolated to other clinical situations, including more acute care, for example, in which the doctor–patient–family dynamic might be different.

This study had its limitations. The short period of time in clinic provided a very limited experience. This study was not designed to identify the best way of exposing pre-clinical students to pediatrics; rather, it identifies several merits and limitations of a simple and feasible model that is currently established. Long-term outcomes, including performance in clerkship, as well as career paths are not expected to be influenced by such a brief clinical encounter and were not measured. We recognize that those who participated were a biased group of highly motivated students who signed up to participate on their own time. We attempted to overcome this bias somewhat by comparing each student's post-clinic attitudes to his/her own pre-clinic attitudes. Nonetheless, motivated students potentially gained more from this experience compared to a broader cohort of randomly selected students. Although the invitation to participate was extended to all pre-clinical students, the majority of the students who participated were in first-year, as second-year students seemed to be more occupied with preparation for board examinations. We studied student benefits of a model in which physicians are generally biased towards an interest in pre-clinical medical education. Successful incorporation of such practices into a medical curriculum would require a broad commitment to undergraduate medical education from providers not necessarily accustomed to junior learners.

## Conclusions

This small study identified ways in which a brief clinical encounter with a pediatric patient and his or her family can be a positive experience for a pre-clinical medical student and helps to justify a common practice of pre-clinical shadowing. It is unclear if pre-clinical curricula should be modified to include such programs although it is reassuring that it is feasible and acceptable to families. With further study, this model might prove to be an effective way to ease the transition into clerkships and supplement the didactic knowledge learned in the classroom.
